# Smart article: application of intelligent platforms in next generation biomedical publications

**DOI:** 10.1186/s40200-017-0314-6

**Published:** 2017-08-03

**Authors:** Babak Mohammadi, Marjan Saeedi, Vahid Haghpanah

**Affiliations:** 1 Endocrinology and Metabolism Research Center, Endocrinology and Metabolism Clinical Sciences Institute, Tehran University of Medical Sciences, Dr. Shariati Hospital, North Kargar Ave, Tehran, 14114 Iran; 2 Assistant Professor of Dermatology, Alborz University of Medical Sciences, Shahid Bahonar Hospital, Karaj, Iran; 3 Personalized Medicine Research Center, Endocrinology and Metabolism Clinical Sciences Institute, Tehran University of Medical Sciences, Tehran, Iran

**Keywords:** Augmented reality, Biomedical, Publication, Predictive text

## Abstract

Production of scientific data has been accelerated exponentially though ease of access to the required knowledge is still challenging. Hence, the emergence of new frameworks to allow more efficient storage of information would be beneficial. Attaining intelligent platforms enable the smart article to serve as a forum for exchanging idea among experts of academic disciplines for a rapid and efficient scientific discourse.

An accelerated increase in the amount of scientific data has led to information overload or an explosion. Measures have been proposed to allow more efficient storage of data, but intelligently retrieval is still a major concern.

On the other hand, growing technologies have provided the possibility of saving various data formats including visual, audio, or even tactile, olfactory [1] and recently, taste modalities. Identifying and pulling out relevant information according to the users’ needs is the key concept of smart article. Smart article is an interactive and intelligent version of the paper article conveying more compact information in various formats [2]. Transform of the contents of linked data sources; and relationship extraction is a basic requirement for preparing such articles. But beside of semantic enrichment in the form of annotated text, smart article will benefit a variety of technological domains.

Through augmented reality systems [3] it is possible to make the research environment more imaginable for the reader. The systems produce a composite view: the user and virtual scenes, and augment this combination with additional information.Virtual, and augmented reality facilitates walking through the research setting and seeing the labs, visiting the patients, listening to the researchers’ speak, and even expediting the hospital or clinic. In this way, the user could communicate with the article based on question-answer real-time dialogues, even through a device, like cyber-glasses [4]. Based on this mutual relationship, he could demand for more information or more focus.

For this purpose, natural language processing [5] is crucial for making an article smart and interactive. This is the ability of computer software to understand human speech. Human speech can be ambiguous and the terminology may be context-related. But the context of a biomedical article can be extracted via text mining [6] and machine learning operations. In addition, it should be perceived that the interaction is scientific and rational, and not profound philosophical or colloquial. The ability of the article to process natural language will support the use of expert systems in the interaction between the smart article and the user.

Expert systems perform the duties that would otherwise be performed by a human. For example, these systems can select and schedule the delivery of extra information. It is possible for an expert system to investigate the user’s questions and assess his level of expertise. Then, the amount and the content of additional requested information is selected according to the questions, or even the position and qualification of the user. This prevents the delivery of improper details and subsequent information overload. Infact, the expert system is the milestone in making a smart article. Sophisticated knowledge-based or rule-based expert systems can be enhanced by gaining additional knowledge or rule, according to the results of data mining. So, these systems could be flexible and dynamic. If the context of an article be determined, the application of other artificial intelligence technologies is possible.

Predictive text is another interesting application of intelligent systems. This technology facilitates typing by suggesting words based on the first letters typed, and therefore provides time economy for the reader and increase the level of interactivity.

Integration of various technologies into an intelligent article could provide an online facility of crowdsourcing. In crowdsourcing a problem is introduced to a population, and the answer is provided by a large network of creative people in the form of collective intelligence. The process may rapidly culminate into scientific progress or even into a paradigm shift. In other words, the article cause the formation of a network of scientists who has experienced the problem by themselves via multimodal channels of information.

Attaining such possibilities enables the smart article to serve as a forum for exchanging idea among experts of academic disciplines to a rapid and efficient scientific discourse. Overall, smart articles provide cognitive enrichment of scientific texts. We believe that the cognitive enrichment would have the potential of changing models of reasoning, training, and practicing medicine.

Medicine has witnessed several paradigm shifts: opinion–, pathophysiological–, and finally, evidence–based hegemony. At the age of dazzling advances in information technology and medical informatics, the emergence of a new era seems inevitable. Smart article, by advancing the frontiers, is capable of playing the role in order to shape the field into a more logical and individually–based medicine.
